# Defining Foreign Patients as ‘Visitors’ and ‘Residents’ in Japanese Medical Facilities: Difficulties in the Collection of Adequate Data

**DOI:** 10.2188/jea.JE20210288

**Published:** 2022-02-05

**Authors:** Soichiro Saeki, Kaori Minamitani, Isao Muraki, Tomoko Shingaki, Koji Nagura, Ken Nakata, Hiroyasu Iso

**Affiliations:** 1 Faculty of Medicine, School of Medicine, Osaka University, Osaka, Japan; 2 Department of Global and Innovative Medicine, Graduate School of Medicine, Osaka University, Osaka, Japan; 3 Department of Public Health, Graduate School of Medicine, Osaka University, Osaka, Japan; 4 Department of International Medical Care, Rinku General Medical Center, Osaka, Japan

We praise the insights of Ishii et al^1^ to differentiate foreign patients from Japanese patients using their names. In addition, we would like to point out that when acquiring data of foreign patients, they should be divided into ‘residents’ and ‘visitors’, such as tourists, because foreign patient issues have been treated as a part of migrant health issues in the world.^2^

Over the last 2 decades in Japan, the number of temporary foreign visitors has increased dynamically to about nine-fold, compared with an approximately 25% increase for residents.^3^ In 2019, although most foreign patients were from non-English countries, only a fifth of hospitals had translation devices, the minimal equipment for communication.^4^ Healthcare issues have increased between hospitals and foreign patients because the Japanese healthcare system reform has been slow in accommodation of foreign patients.^5^ There are several differences in clinical decision-making and healthcare needs between resident and visitor foreign patients. Visitors, who hope to return to their homeland, can have limited treatment options in acute care (for example, the restriction from long-term hospitalization) because of their healthcare insurance or treatment options in their homeland. For residents, chronic care can be more important than acute care. However, foreign patient issues are not limited to medical treatment. Less than 10% of residents and around 30% of visitors could not afford their medical expenses in 2019.^4^ Therefore, the identification of visiting status in foreign patients is essential to interpret their healthcare contexts in Japan. To identify the visiting status of foreign patients using routinely collected hospital records, we examined if patients’ data on addresses and type of healthcare insurance would be feasible to group foreign patients into residents and visitors.

From the electronic health records from January 2011 through March 2018, we identified 692 patients using medical communication support due to limited Japanese proficiency in the Rinku General Medical Center (Izumisano, Osaka, Japan), which is located very close to the Kansai International Airport and accepts many foreign patients from throughout Osaka.^6^ Data of patients’ features, such as healthcare insurance used for initial payment and residency, was obtained, and patients were classified as residents or visitors accordingly (Table 1). The study protocol was approved by the Ethics committee of the Rinku General Medical Center (approval number 20-041).

**Table 1.** Criteria of grouping patients into visitors or residents

Criteria	Visitors	Residents	Unknown
**Address**	Patients staying in an address for accommodation facilities such as hotels or address outside of Japan.	Patients with recorded addresses not matched with the visitor criteria.	Address not provided

**Insurance**	Patients that paid full terms of medical expense by themselves.	Patients that paid with the National Health Insurance, employee’s health insurance, medical aid for welfare recipients, and the Industrial Accident Compensation Insurance issued under the Japanese insurance system.	Patients receiving healthcare checkups, such as *Ningen Dock*, or patients covered by insurance from traffic accidents, or with insurance information unprovided

Of the 579 patients remaining after excluding those having unknown data in either of residency and insurance (*n* = 113), 292 residents and 178 visitors were concordantly identified by both residency and insurance, but 109 patients were discordant (kappa statistic = 0.618, Table 2). Among 470 concordant patients, the most visited department was different between residents and visitors: although only 7.9% of resident patients visited the department of emergency, trauma, or critical care, 57.9% of visitor patients visited there (*P* < 0.001 using chi-squared test, Table 3). To determine the reason for discordance in identification methods, we reviewed individual hospital records of the 109 discordant patients. Around half of the 100 discordant patients with Japanese residency visited obstetrics and gynecology or pediatrics, possibly due to medical procedures not covered by the Japanese health insurance system, including prenatal care, normal delivery, and vaccination.^7^ The rest were short-term overseas students, homestay students, or family members visiting their relatives having Japanese residency. The discordant reason for nine patients without Japanese residency was unclear from their hospital records.

**Table 2.** Classification of foreign patients into visitors or residents by address and insurance

	Address			Total

	Residents	Visitors	Unknown	
Insurance				
Residents	292	9	0	301
Visitors	100	178	0	278
Unknown	68	44	1	113
Total	460	231	1	692

**Table 3.** First visited department of resident and visitor foreign patients

	Residents (*N* = 292)		Visitors (*N* = 178)		*P*-value
Internal medicine	112	(38.4%)	22	(12.4%)	<0.001
Obstetrics and gynecology	54	(18.5%)	9	(5.1%)	<0.001
Pediatrics	29	(9.9%)	13	(7.3%)	0.33
Emergency, trauma, and critical care	23	(7.9%)	103	(57.9%)	<0.001
Otorhinolaryngology/Head & Neck Surgery	17	(5.8%)	7	(3.9%)	0.37
Urology	17	(5.8%)	6	(3.4%)	0.23
Surgery	17	(5.8%)	1	(0.6%)	0.003
Orthopedics	9	(3.1%)	4	(2.2%)	0.77
Plastic Surgery	6	(2.1%)	4	(2.2%)	1.00
Oral Surgery	5	(1.7%)	1	(0.6%)	0.42
Ophthalmology	2	(0.7%)	0	(0.0%)	0.53
Neurosurgery	1	(0.3%)	7	(3.9%)	0.006
Dermatology	0	(0.0%)	1	(0.6%)	0.38

*P* values were calculated using Chi-square test for items with five or more in each group and using Fisher’s exact test for those with less than five in either group.

Our findings demonstrate that resident patients and visitor patients may have some differences in healthcare needs and clinical conditions. However, neither address nor insurance in electronic health records can accurately group foreign patients into residents and visitors. To acquire accurate statistics of healthcare use among foreign patients in Japan, we propose that, in addition to the information for the standardized identification methods of foreign patients, their visiting status should be obtained from official documents, including their residence card or visa types.
